
JOURNAL INFORMATION
==============================
Journal ID: Wirtschaftsdienst

Wirtschaftsdienst

EISSN: 1613-978X

ARTICLE INFORMATION
==============================
PMCID: PMC9226268
DOI: 10.1007/s10273-022-3205-5
Article ID: 3205
Article version: 1
Subjects: Kommentare

Sozialstaat: Daseinsvorsorge stärken!

Welfare state: Strengthening services of general interest!

Butterwegge Christoph butterwegge-politikwissenschaft@uni-koeln.de

grid.6190.e 0000 0000 8580 3777 Inst. f. Vergleichende Bildungsforsch. u. Sozialw., Universität zu Köln, Gronewaldstr. 2, 50931 Köln, Deutschland
Electronic publication date: 2022 Jun 24
Print publication date: 2022
Volume: 102
Issue: 6
First page: 421
Last page: 421
Copyright: © Der/die Autor:in 2022
License: Open Access: Dieser Artikel wird unter der Creative Commons Namensnennung 4.0 International Lizenz veröffentlicht (https://creativecommons.org/licenses/by/4.0/deed.de).
Open Access wird durch die ZBW — Leibniz-Informationszentrum Wirtschaft gefördert.
License URL: https://creativecommons.org/licenses/by/4.0/

Keywords: D62, D63, I18
issue-copyright-statement: © The Author(s) 2022

==============================

Wenn eine Epidemie den Charakter einer Gesellschaft offenbart, hat Deutschland seine Bewährungsprobe während der Coronakrise mitnichten bestanden. Vielmehr hat die Pandemie bestehende Ungleichheiten eher verstärkt, ohne dass hieraus Konsequenzen gezogen wurden. Corona war aber mit Sicherheit weder die erste noch die letzte Pandemie, sondern im Zeitalter existenzieller Krisen möglicherweise nur der Beginn einer Serie sozial-, gesundheits-, wirtschafts- und finanzpolitischer Herausforderungen dieser Größenordnung. Ein teilprivatisiertes, gewinnorientiertes Gesundheitssystem garantiert keine optimale medizinische Behandlung der Kranken und in Notsituationen wie einer Pandemie auch keine maximale Versorgungssicherheit für die Bevölkerung. Eine der wichtigsten Lehren, die aus der Pandemie gezogen werden müssen, ist deshalb die Notwendigkeit eines leistungsfähigen, nicht durch Ökonomisierung, Privatisierung und Kommerzialisierung geschwächten Gesundheitssystems. Krankenhäuser, die sich immer stärker zu profitorientierten Gesundheitsunternehmen entwickelt haben, sollten wieder zu bedarfsgesteuerten Institutionen der öffentlichen Daseinsvorsorge werden. Klinikkonzerne wie Helios, Sana und Asklepios sind teilweise börsennotierte Unternehmen, die sich vorrangig um eine optimale Verwertung des eingesetzten Kapitals bemühen und maximale Renditen erwirtschaften müssen. Sie richten ihr Hauptaugenmerk dabei weder auf das Wohl des medizinischen Personals und der Pflegekräfte noch auf das der von diesen behandelten Patient:innen.

Als die COVID-19-Pandemie Deutschland erfasste, hat sich der Sozialstaat als „systemrelevant" und im Kern als funktionstüchtig erwiesen. Ohne Leistungen wie das Kurzarbeitergeld wären noch mehr Familien während des Lockdowns und der teilweise darauf zurückzuführenden Rezession an den Rand des Ruins geraten. Auch war es richtig, dass der Arbeitslosengeld-II-Bezug für von der Coronakrise gebeutelte Soloselbstständige erleichtert wurde, indem man für sie die strenge Vermögensprüfung aussetzte und die Angemessenheit der Wohnung stillschweigend voraussetzte. Warum sollte diese Regelung keine Dauerlösung und nicht das Vorbild für weitere Schritte zur Entbürokratisierung des Sozialstaats und zur Vereinfachung des Antragsverfahrens in der Grundsicherung sein? Zugangsbeschränkungen, Strukturdefizite und Leistungshemmnisse des Sozialstaats sind während der Pandemie ebenfalls klarer zutage getreten. Obwohl auch Kinderlose pandemiebedingt erhöhte Ausgaben hatten, weil viele Tafeln geschlossen, preiswerte Lebensmittel wegen Hamsterkäufen eher Mangelware und Desinfektionsmittel teuer waren, stellte sich die Regierung bei der Forderung nach einer vorübergehenden Erhöhung von Hartz IV taub. Jobcenter weigerten sich, die Anschaffung digitaler Endgeräte für Kinder im Homeschooling als Sonderbedarf der Grundsicherung anzuerkennen. Ebenfalls abgelehnt wurde die Übernahme der Kosten für PCR-Tests.

Kleinstrentner:innen und Studierende die wegen des Lockdowns sowie vieler Geschäftsaufgaben und Betriebsschließungen ihren Minijob verloren, konnten weder Kurzarbeiter- noch Arbeitslosengeld (I bzw. II) beantragen. Hieraus sollte der Schluss gezogen werden, dass Minijobs in sozialversicherungspflichtige Beschäftigungsverhältnisse umgewandelt werden müssen. Man kann geradezu von einer Fehlkonstruktion der staatlichen Finanzhilfen sprechen, die sich nicht am Bedarf der ärmsten Bevölkerungsgruppen orientieren. Statt des Leistungsprinzips, das ökonomischen Erfolg prämiert, muss das Bedarfsprinzip als Hauptkriterium im Mittelpunkt aller Hilfsmaßnahmen stehen und die Maxime lauten: Wer wenig hat, muss besonders viel, und wer viel hat, muss entsprechend wenig Unterstützung vom Sozialstaat bekommen. Menschen, die durch alle Maschen des Systems der sozialen Sicherung fallen, dürfen nicht in existenzielle Bedrängnis geraten. Nötig wäre eine bedarfsgerechte Konzentration staatlicher Ressourcen auf Personen, die Unterstützung benötigen, um in Würde leben zu können. Das gilt für prekär Beschäftigte, Leiharbeiter:innen und Randbelegschaften ebenso wie für Soloselbstständige, Freiberufler:innen und Kleinunternehmer:innen, die nicht über finanzielle Rücklagen verfügen.

Die zentrale Lehre aus der Pandemie lautet, nicht länger neoliberalen Verlockungen à la „privat geht vor Staat" zu erliegen. Statt prioritär auf den Markt zu setzen, sollte man die öffentliche Daseins- und Gesundheitsvorsorge stärken. Es gibt einen solidarischen Weg aus der Coronakrise: Wenn der Wohlfahrtsstaat umfassender für einen Ausbau der sozialen, Bildungs- und Betreuungsinfrastruktur sorgt und mehr Investitionen tätigt, kann die Gesellschaft sogar im äußersten Notfall funktionsfähig bleiben, die damit verbundenen Probleme bewältigen und ihre besonders gefährdeten Mitglieder optimal schützen.


Literatur

Butterwegge, C. (2022), Die polarisierende Pandemie: Deutschland nach Corona, Beltz Juventa.
